# CHRONIC DISEASE SELF-MANAGEMENT PROGRAM ATTENDANCE AMONG AFRICAN AMERICANS WITH ARTHRITIS AND COMORBIDITIES

**DOI:** 10.1093/geroni/igz038.268

**Published:** 2019-11-08

**Authors:** Chivon A Mingo, Tiffany R Washington, Matthew L Smith

**Affiliations:** 1 Georgia State University, Atlanta, Georgia, United States; 2 University Of Georgia, Athens, Georgia, United States; 3 Texas A&M University, College Station, Texas, United States

## Abstract

African Americans (AA) are 17% less likely to be diagnosed with arthritis compared to Whites, yet disproportionately burdened by arthritis symptoms. AA are overrepresented in the diagnosis and burden of other chronic conditions (diabetes, heart disease), highlighting the need to engage them in evidence-based Chronic Disease Self-Management Education (CDSME) Programs. This study examines how disease profiles may influence program attendance. Using a multinomial logistic regression, data were analyzed from AA with arthritis (N=20,541) who attended CDSME programs in 48 states. Relative to those with only arthritis, participants with more complex disease profiles were less likely to attend an arthritis-specific program (P<0.001) and more likely to attend a diabetes-specific program (P<0.001). Those with more complex disease profiles were more likely to attend programs at healthcare organizations and residential facilities, and less likely to attend in faith-based organizations (P<0.001). Understanding barriers and facilitators to program attendance have policy and public health implications.

